# Supplementary material for: Causal network models of SARS-CoV-2 expression and aging to identify candidates for drug repurposing
Source: Nat Commun. 2021 Feb 15;12:1024. doi: 10.1038/s41467-021-21056-z (PMC7884845; doi:10.1038/s41467-021-21056-z)
Supplement: Supplementary file 1 — Supplementary Information [file 41467_2021_21056_MOESM1_ESM.pdf]

Supplementary Materials: Causal Network Models of SARS-CoV-2 Expression and Aging Identify Drugs for Repurposing

Anastasiya Belyaeva[1#], Louis Cammarata[2#], Adityanarayanan Radhakrishnan[1#], Chandler Squires[1], Karren Dai Yang[1], G.V. Shivashankar[3,4], Caroline Uhler[1*]

[1]Massachusetts Institute of Technology, U.S.A.
[2]Harvard University, U.S.A.
[3]ETH Zurich, Switzerland
[4]Paul Scherrer Institute, Switzerland

[#]Equal contribution.
[*]To whom correspondence should be addressed; E-mail: cuhler@mit.edu.

This PDF file includes:

Supplementary Note
Supplementary Figures 1-24
Supplementary Tables 1-2
Supplementary References

Supplementary Note

Overview of methodology

Our drug discovery pipeline consists of three parts: mining relevant drugs, identifying the disease interactome, and investigating the drug mechanism. Fig. 1 describes the inputs, outputs and algorithms used in each of the three parts. Briefly, the first part (mining relevant drugs) takes in normal and infected/diseased RNA-seq samples along with the public CMap database, which contains gene expression data on cell lines treated with a variety of FDA approved compounds, to train an autoencoder and subsequently construct synthetic interventions in the learned latent space. It outputs a list of drugs ranked by the correlation of each drug with the reverse disease signature. The second part of the pipeline (identifying disease interactome) also takes in the normal and infected/diseased RNA-seq samples as well as a PPI network (e.g. from the public IREF or STRING databases). It then identifies the genes that are differentially expressed in the disease and learns the disease interactome connecting these genes in the PPI network using the prize-collecting Steiner forest algorithm. In addition, the inferred ranked list of drugs output from part 1 in the pipeline is mapped to its targets using the public DrugCentral database. The drug targets are intersected with the disease interactome to further filter the list of drugs to only include those drugs that target nodes in the interactome. The third part of the pipeline (investigating drug mechanism) uses multi-sample RNA-seq data (e.g. high number of replicates or single-cell RNA-seq data) to learn the causal directions in the disease interactome using GSP, a causal structure discovery algorithm, and identifies which drugs and drug targets have the largest downstream causal effect on the disease interactome.

Comparison of SARS-CoV-2 versus IAV and RSV

In order to test how specific our findings are to SARS-CoV-2 and demonstrate the broad applicability of our pipeline, we apply our computational pipeline to two additional viral infections: respiratory syncytial virus (RSV) and influenza A virus (IAV). As for SARS-CoV-2 infection, we obtain gene expression data for these viruses from [1]. First, we perform differential expression analysis for IAV and RSV (Supplementary Fig. 20) showing that only 3.19% and 19.6% of genes specific to SARS-CoV-2 are shared with RSV and IAV, respectively. Next, we apply our over-parameterized autoencoder and synthetic interventions framework to IAV and RSV to obtain drug lists ranked by their correlation with the reverse disease signature.

In order to quantitatively compare the drug lists obtained for RSV and IAV to the drug list for SARS-CoV-2, we measure the similarity of two rankings using curves akin to a receiver operating characteristic (ROC) curve, namely: given two rankings of n drugs, we consider the top k drugs in one of the lists and compute the number of drugs in common among these top k drugs for $k = 1, 2, \dots n$. Thus, the x-coordinate in each plot indicates the proportion, k/n, of each drug list we consider and the y-coordinate is the size of the intersection of the two subsets normalized by k. The area under the curve (AUC) is a measure of similarity between two drug lists. When two drug lists are exactly the same, the AUC is 1 and when the two drug lists are maximally different (i.e., one drug list is the reverse of the other), the AUC is $1 - \ln(2) \approx .306$; see Supplementary Fig. 9a. Supplementary Fig. 21a-b show that that the drug lists for SARS-CoV-2 and RSV are significantly different and in fact very close to the lower bound, while the drug lists for SARS-CoV-2 and IAV are quite similar with an AUC of 0.843.

Finally, we perform the Steiner tree analysis based on the identified differentially expressed genes for IAV and RSV as well as the drug lists obtained by the overparameterized autoencoder. As for SARS-CoV-2, since the morbidity and fatality rate of IAV is higher in the aging population, we compute a combined IAV and aging interactome. This consists of 185 nodes and 486 edges based on 124 terminal genes. Since RSV is riskier in young children, but can also be serious for the aging population, we compute two interactomes, one without taking aging into account (234 nodes and 871 edges based on 139 terminal genes) and one combined with RSV and aging (303 nodes and 1177 edges based on 200 terminal genes) to make it more comparable to the other interactomes. To make the results comparable, since for SARS-CoV-2 we intersected the targets of the top 142 ranked drugs from the overparameterized autoencoder analysis with the interactome, we perform the analysis with the same number of drugs also for IAV and RSV. The resulting drugs and drug targets are shown in Supplementary Fig. 22. For IAV, this results in 20 drugs, 13 of which overlap with drugs identified in the SARS-CoV-2 analysis. These drugs target 9 proteins in the interactome, 2 of which are also present in the SARS-CoV-2 interactome, namely EGFR and RIPK1. For RSV with and without aging the resulting drug lists as well as their

targets have no overlap with the ones identified by SARS-CoV-2. In particular, the identified drug lists contain no tyrosine kinase inhibitors, thereby indicating the specificity of our results to SARS-CoV-2.

Randomization analysis

(1) Randomization of PPI network: Randomization of the IREF protein-protein interaction network was performed via randomly permuting the vertex labels. Such randomization affects a gene's neighborhood while preserving basic network properties such as number of edges and degree distribution. The prize-collecting Steiner tree analysis pipeline was then applied to this new network. Drugs targeting terminal nodes were systematically selected in all randomization runs, as expected given that the prize-collecting Steiner tree algorithm parameters were set so that all terminal nodes are included in the solution. Other drugs identified by the non-randomized analysis that did not target any terminal node appeared with frequencies varying from 56% (primaquine, which has 5 targets in the network) to 97% (imatinib, which has 69 targets in the network). Only two drugs (mifepristone and palbociclib) that were not selected by the non-randomized analysis appeared more frequently (80% of runs) than the least frequently selected drug from the non-randomized analysis (primaquine, 56% of runs).

(2) Permuting expression data: Randomizing gene labels in the RNA-seq expression data set from [1] while preserving gene labels of the GTEx aging data set is an implicit approach to randomizing the list of terminal genes used as input for the prize-collecting Steiner tree algorithm. After applying the Steiner tree analysis pipeline, the drugs selected in the non-randomized analysis appeared between 18% (milrinone) and 100% (sunitinib) of the runs. Generally, the more proteins a drug targeted in the IREF network, the more frequently it appeared in the solution (sunitinib, with 260 targets, is the drug with highest number of targets in the PPI network). 16 drugs that were not selected in the non-randomized analysis (this represents 1% of the set of non-selected drugs) appeared more frequently than the least frequently selected drug from the non-randomized analysis (milrinone).

(3) Randomization of CMap signatures: We also ran the Steiner tree analysis after randomly permuting the SARS-CoV-2-anticorrelation scores of the 605 CMap drugs and selecting the drugs with anticorrelation above 0.86 (resulting in 142 drugs as in the original non-randomized analysis). After applying the Steiner tree analysis pipeline, drugs that were selected in the non-randomized analysis appeared in the final list with a frequency between 22% and 26%, as expected (since $142/605 \approx 23.5\%$). More interestingly, 17 drugs which were not selected in the non-randomized analysis (representing 1% of the overall set of non-selected drugs) appeared at a similar 22-29% frequency in the solution. These are drugs that target one of the network nodes yet have a true SARS-CoV-2-anticorrelation score lower than 0.86.

(4) Randomization of terminal nodes: Finally, we directly randomized the list of terminal nodes, by randomly selecting 162 genes from the RNA-seq expression dataset and prizing them with their corresponding absolute $\log_2$ fold change after SARS-CoV-2 infection in A549-ACE2 cells. The drugs selected in the non-randomized analysis appeared between 3% (milrinone) and 100% (sunitinib) of the runs. In this analysis, 41 drugs that were not selected in the non-randomized analysis (this represents 2.5% of the set of non-selected drugs) appeared more frequently than the least frequently selected drug from the non-randomized analysis (milrinone).

These results show that while the output of our Steiner tree analysis pipeline is quite robust to changes in the underlying PPI network, the selection of the terminal nodes has a critical effect on the final drug list.

Supplementary Figures

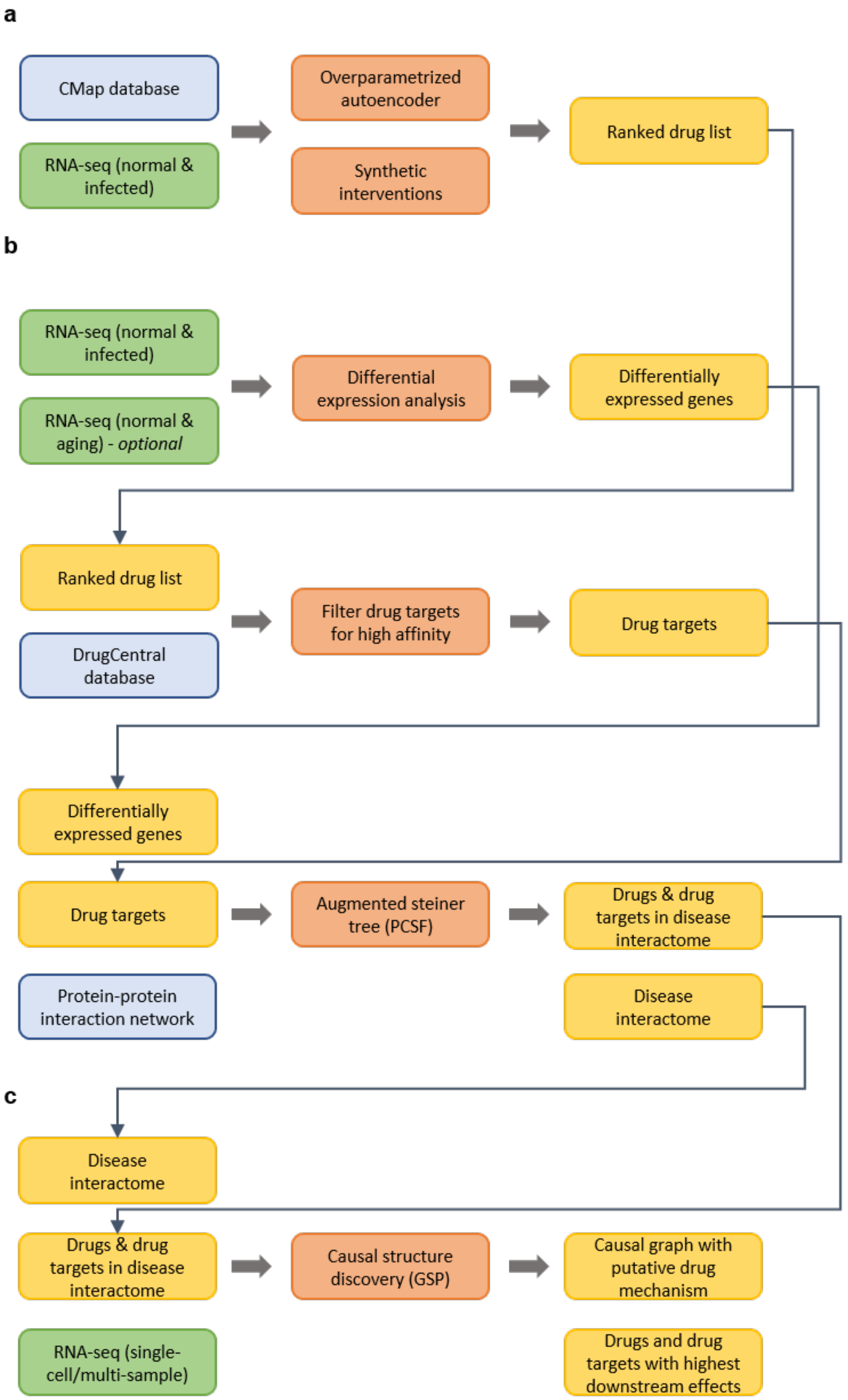

Supplementary Fig. 1: Detailed schematic of our computational drug repurposing platform. Green boxes denote inputs that may need to be collected for the specific virus/disease and cell type of interest. Blue boxes denote inputs corresponding to databases that are publicly available. Orange boxes denote our computational methods and yellow boxes denote method outputs. Computational pipeline for (a) mining relevant drugs, (b) identifying disease interactome and (c) investigating drug mechanism.

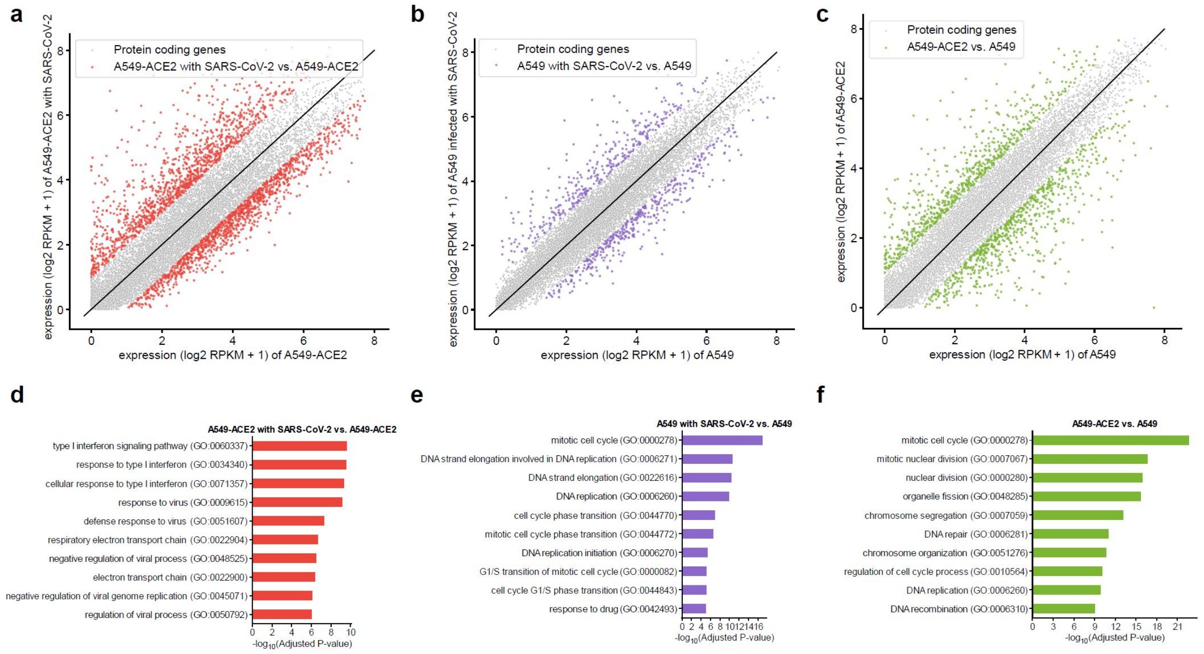

Supplementary Fig. 2: (a) Gene expression of A549-ACE2 cells with and without SARS-CoV-2 infection, with differentially expressed genes in red. (b) Gene expression of A549 cells with and without SARS-CoV-2 infection, with differentially expressed genes in purple. (c) Gene expression of A549 cells with and without ACE2 receptor, with differentially expressed genes in green. (d) Top 10 gene ontology terms associated with differentially expressed genes between A549-ACE2 cells with and without SARS-CoV-2 infection. (e) Top 10 gene ontology terms associated with differentially expressed genes between A549 cells with and without SARS-CoV-2 infection. (f) Top 10 gene ontology terms associated with differentially expressed genes between A549 cells with and without ACE2 receptor. All gene ontology terms have adjusted p-value < 0.05 (adjusted for multiple hypothesis testing using Benjamini–Hochberg procedure).

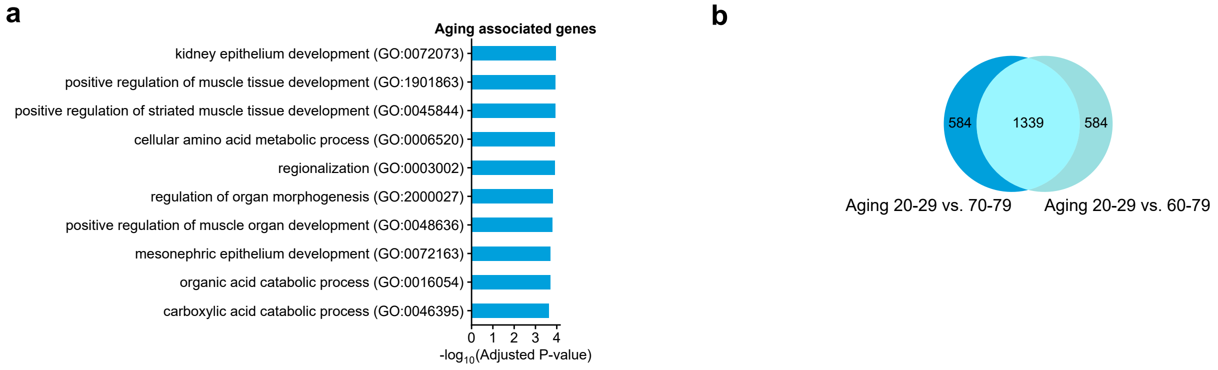

Supplementary Fig. 3: (a) Top 10 gene ontology terms associated with aging (adjusted for multiple hypothesis testing using Benjamini–Hochberg procedure). (b) Venn diagram showing significant overlap between aging associated genes considering different definitions of older, specifically just individuals in the oldest category of 70-79 years old (dark blue, left circle) or individuals that are 60-79 (light blue, right circle).

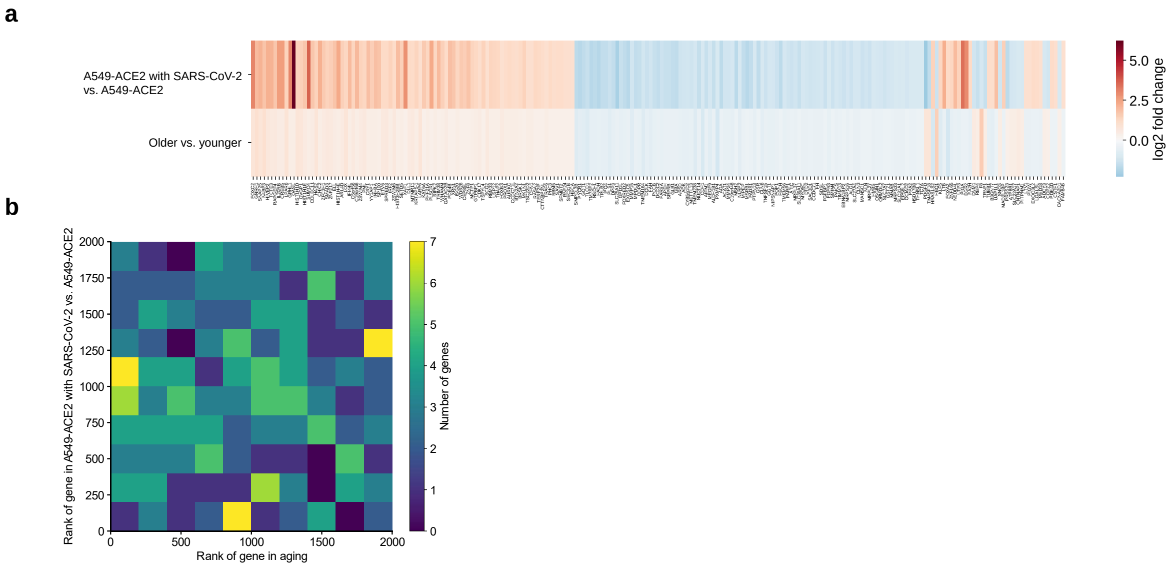

Supplementary Fig. 4: (a) Heatmap of $\log_2$-fold changes of differentially expressed genes shared by SARS-CoV-2 and aging with gene names. (b) 2D histogram of the number of genes having a certain rank in aging and SARS-CoV-2 datasets.

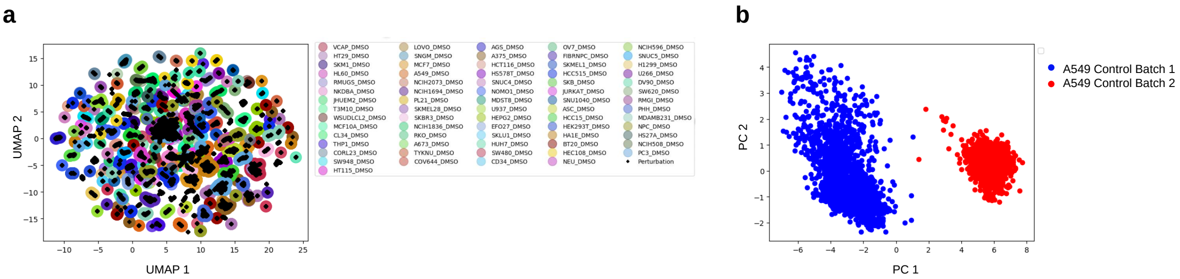

Supplementary Fig. 5: (a) Uniform Manifold Approximation and Projection (UMAP) of control and perturbations across all cell types in Connectivity Map (CMap). The effect of a perturbation (black) on a given cell type is small relative to the differences between cell types (cell types are denoted by different colors). (b) Principal component analysis highlighting batch effects for the control samples of the A549 cell line from CMap. K-means clustering by gene expression vector is used to identify and remove batch effects (represented as red and blue clusters).

a

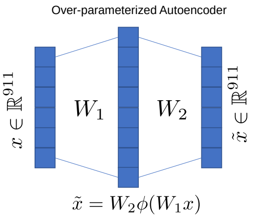

b

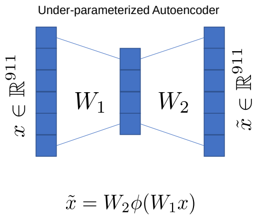

c

Num. Hidden Units	Num. Hidden Layers	Nonlinearity	Optimizer, LR	Initialization	Seed Used	Training Loss	Test Loss
1024	1	Leaky ReLU	Adam, 1e-4	PyTorch Default	17	7.3 x 10^-7	1.1 x 10^-6
100	1	Leaky ReLU	Adam, 1e-4	PyTorch Default	17	2.8 x 10^-3	2.8 x 10^-3
1024	1	CosID	Adam, 1e-4	PyTorch Default	17	6.4 x 10^-6	6.5 x 10^-6

Supplementary Fig. 6: Overview of autoencoder architectures, optimization methods and hyperparameter settings considered. (a) Diagram representing an overparameterized autoencoder. While this autoencoder is capable of learning the identity function, training leads to a solution that better aligns drug signatures across cell types in the latent space. (b) Diagram representing an underparameterized autoencoder. While this architecture is most commonly used in practice, it does not align drug signatures as well in the latent space as its overparameterized counterpart; see Supplementary Fig. 8. (c) Details on the width, depth, nonlinearity, optimization method, learning rate, random seed, training loss and test loss for all architectures considered in this work.

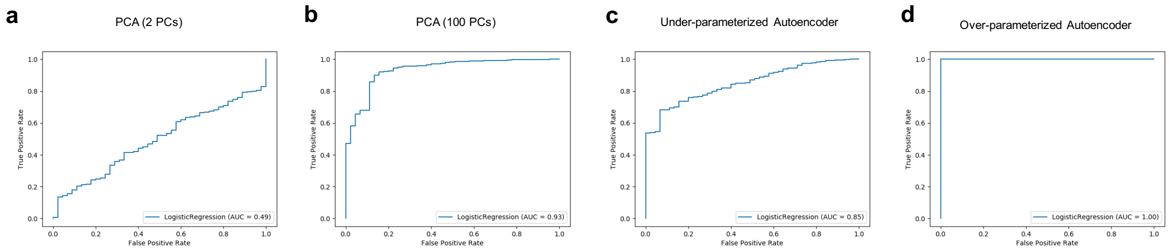

Supplementary Fig. 7: Receiver operating characteristic (ROC) curves for the agreement in classification between gene expression vectors and reconstructed gene expression vectors obtained using an embedding given by the first 2 principle components in (a), the first 100 principle components in (b), an underparameterized autoencoder in (c), and an overparameterized autoencoder in (d). While a logistic regression model trained to classify between 831 A549 control samples and 32893 A549 perturbation samples shows differences in predictions on original gene expression vectors versus underparameterized autoencoder reconstructions and reconstructions from the top 2 or 100 principal component, the overparameterized embedding allows near perfect reconstruction of the original gene expression vectors with no difference in predictions between using overparameterized embeddings for gene expression vectors and original gene expression vectors.

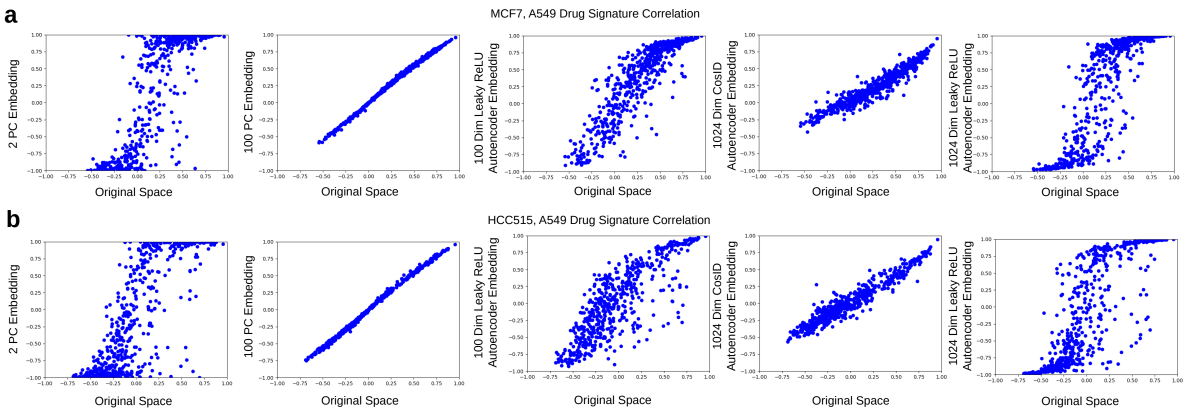

Supplementary Fig. 8: Comparison of drug signature alignment between A549 and MCF7 (top) and A549 and HCC515 (bottom) cell types upon using an embedding verus the original space. Embeddings provided include (from left to right) top 2 PCs, top 100 PCs, underparameterized leaky ReLU autoencoder, overparameterized cosid autoencoder, overparameterized leaky ReLU autoencoder. Embeddings from the overparameterized autoencoder with leaky ReLU activation better align drug signatures between these two pairs of cell types than any other embedding considered while still providing near perfect reconstruction of the original data.

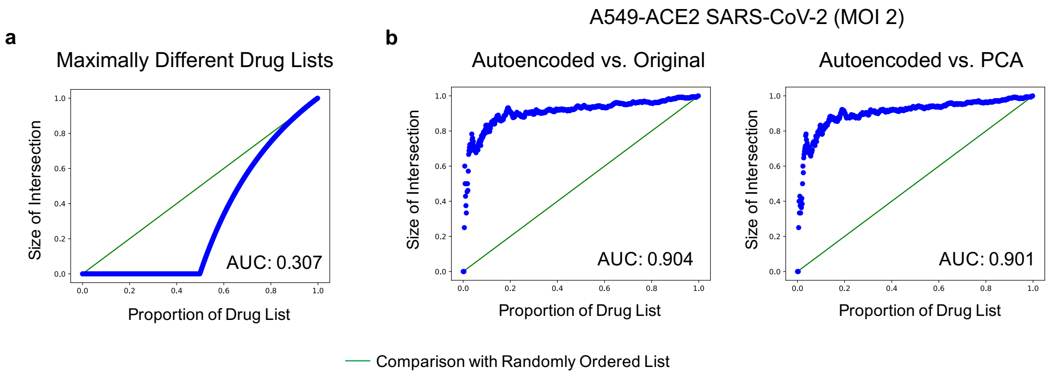

Supplementary Fig. 9: Quantitative analysis of similarity between drug lists obtained using the latent space embedding as compared to the original and PCA embedding (using 2 PCs). Given two rankings of n drugs, we consider the top k drugs and plot the number of drugs in common among these top k drugs for $k = 1, 2, \dots n$; i.e., the x-coordinate of a point indicates the proportion, k/n, of each drug list we consider and the y-coordinate is the size of the intersection of the two subsets normalized by k. AUC denotes the area under the curve; green line indicates the expected size of intersection for randomly chosen lists; (a) shows the result when considering two maximally different drug lists, i.e., when one is the reverse of the other, resulting in an AUC of 0.307; (b) demonstrates that the drug list produced in the latent space of the over-parameterized autoencoder is similar to that produced in the original space and to that produced using 2 PCs. The advantages of using the over-parameterized autoencoder are that the resulting latent space contains enough signal to reconstruct gene expression vectors well and provides better alignment between drug signatures across cell types than in the original space.

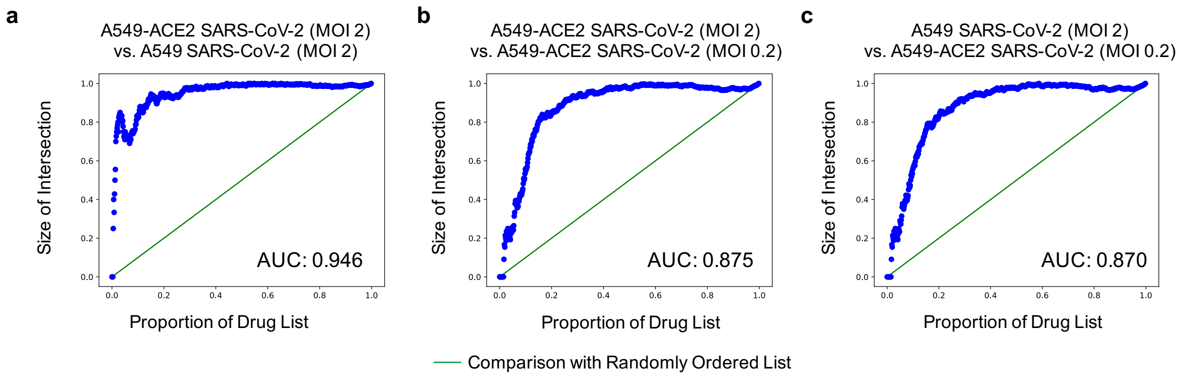

Supplementary Fig. 10: Quantitative analysis of similarity between drug lists obtained using the overparameterized autoencoder on gene expression data from different MOIs for A549 cells with and without ACE2 supplement. (a) Comparison of drug lists obtained from SARS-CoV-2 infected A549-ACE2 cells with MOI 2 and A549 cells with MOI 2, (b) A549-ACE2 cells with MOI 2 and A549-ACE2 cells with MOI 0.2, and (c) A549 cells with MOI 2 and A549-ACE2 cells with MOI 0.2. The similarity between the drug lists drops when comparing an MOI of 2 to an MOI of 0.2, which is consistent with the observation by [1] that low-MOI conditions did not stimulate an important interferon-I and -III response.

a

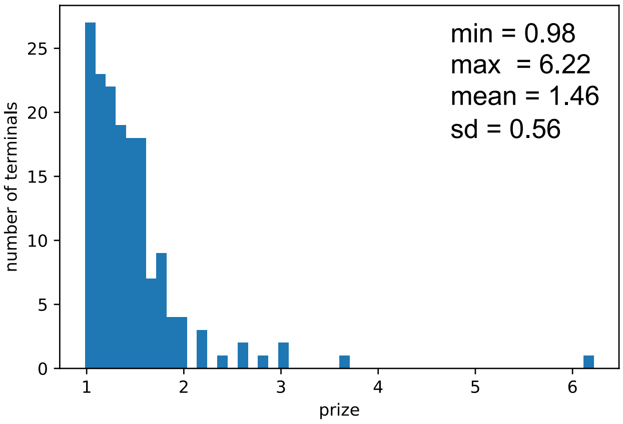

b

75 upregulated terminals

gene	prize	log2FC virus	log2FC age
OASL	6.22	6.22	0.50
SUMO4	3.64	3.64	0.48
GRHL1	3.01	3.01	0.51
FOXC2	3.00	3.00	0.81
XAF1	2.84	2.84	0.39
IL20RB	2.62	2.62	0.53
CREB5	2.59	2.59	0.53
PLSCR1	2.36	2.36	0.36
CHIC2	2.22	2.22	0.49
HIVEP2	2.21	2.21	0.69
SNAP25	2.18	2.18	0.82
C19ORF66	2.01	2.01	0.46
GNRH1	1.99	1.99	0.99
TRIM38	1.96	1.96	0.37
HIST3H2BB	1.91	1.91	0.42
PDE4B	1.90	1.90	0.37
CRY1	1.87	1.87	0.46
INHBA	1.85	1.85	0.45
ZNF8	1.80	1.80	0.36
SP100	1.79	1.79	0.44
N4BP3	1.77	1.77	1.04
ELL	1.74	1.74	0.51
RAPGEF4	1.73	1.73	0.67
XRN1	1.72	1.72	0.38
HIST1H1E	1.71	1.71	0.56
WDR26	1.66	1.66	0.38
ZFC3H1	1.66	1.66	0.55
KAT6A	1.60	1.60	0.41
CEP85L	1.57	1.57	0.39
YY1AP1	1.52	1.52	0.48
ZNF217	1.51	1.51	0.59
OVGP1	1.48	1.48	0.77
SETD5	1.48	1.48	0.46
ARL4D	1.47	1.47	0.57
IRF2	1.45	1.45	0.47
SMAD3	1.45	1.45	0.67
HIST1H1D	1.44	1.44	0.84
TSPYL4	1.44	1.44	0.40
MKLN1	1.43	1.43	0.35
HAS2	1.40	1.40	0.82
RBM33	1.40	1.40	0.41
ISG20	1.36	1.36	0.45
USP12	1.35	1.35	0.42
LOX	1.34	1.34	0.78
PHF21A	1.33	1.33	0.48
RIPK2	1.33	1.33	0.47
GTPBP1	1.30	1.30	0.46
PML	1.30	1.30	0.67
RASA2	1.30	1.30	0.37
THAP2	1.28	1.28	0.44
PHC3	1.26	1.26	0.36
BRSK1	1.25	1.25	0.42
DLL1	1.23	1.23	0.57
ETV6	1.23	1.23	0.62
TERF2IP	1.22	1.22	0.38
AURKC	1.21	1.21	0.45
SPRED3	1.21	1.21	0.66
GATAD2B	1.18	1.18	0.52
MTMR11	1.18	1.18	0.60
ZSWIM6	1.18	1.18	0.66
PROX1	1.16	1.16	0.47
CTTNBP2NL	1.15	1.15	0.41
CCDC71L	1.14	1.14	0.46
SAV1	1.13	1.13	0.36
CDK17	1.10	1.10	0.53
RBM5	1.09	1.09	0.38
BTN2A2	1.08	1.08	0.47
TSC22D2	1.07	1.07	0.46
LARP6	1.06	1.06	0.79
RNF24	1.06	1.06	0.37
SEC31B	1.05	1.05	0.36
CHN1	1.04	1.04	0.46
HOXB3	1.01	1.01	0.55
SCG2	1.01	1.01	0.58
TRAF6	1.00	1.00	0.46

87 downregulated terminals

gene	prize	log2FC virus	log2FC age
SLC25A11	2.00	-2.00	-0.46
HADH	1.77	-1.77	-0.52
PYCR1	1.74	-1.74	-0.63
TM7SF2	1.74	-1.74	-0.53
EXOSC5	1.71	-1.71	-0.47
MSRB1	1.70	-1.70	-0.37
MSH2	1.65	-1.65	-0.39
NSDHL	1.63	-1.63	-0.56
SMPDL3B	1.60	-1.60	-0.77
AGA	1.59	-1.59	-0.40
FECH	1.59	-1.59	-0.36
GOT1	1.58	-1.58	-0.71
GBA	1.57	-1.57	-0.44
GSR	1.57	-1.57	-0.38
FOXRED2	1.56	-1.56	-0.49
GLB1	1.54	-1.54	-0.44
TRUB2	1.54	-1.54	-0.54
DCDC2	1.53	-1.53	-0.46
FADS1	1.53	-1.53	-0.40
IPO4	1.53	-1.53	-0.54
DPP3	1.52	-1.52	-0.53
MPDU1	1.51	-1.51	-0.49
MIPEP	1.49	-1.49	-0.41
ALDH1B1	1.48	-1.48	-0.44
QDPR	1.48	-1.48	-0.49
NUDT14	1.46	-1.46	-0.45
GPD1L	1.44	-1.44	-0.55
SRPRB	1.44	-1.44	-0.47
AIFM1	1.42	-1.42	-0.45
TLCD1	1.41	-1.41	-0.83
APEH	1.40	-1.40	-0.48
PCCB	1.39	-1.39	-0.51
SLC39A11	1.39	-1.39	-0.38
DOLK	1.37	-1.37	-0.53
FGFBP1	1.37	-1.37	-0.37
PLS1	1.37	-1.37	-0.68
EBNA1BP2	1.34	-1.34	-0.36
TNFSF15	1.34	-1.34	-0.44
POP1	1.33	-1.33	-0.43
FARSA	1.32	-1.32	-0.52
ALG1	1.30	-1.30	-0.37
ATIC	1.30	-1.30	-0.42
MRPL27	1.29	-1.29	-0.36
SAC3D1	1.28	-1.28	-0.41
METTL13	1.27	-1.27	-0.51
PARP1	1.27	-1.27	-0.44
SLC7A7	1.26	-1.26	-0.36
MMP15	1.25	-1.25	-0.73
TIMM13	1.25	-1.25	-0.40
CCDC71	1.24	-1.24	-0.42
FAH	1.23	-1.23	-0.65
MRPL37	1.23	-1.23	-0.44
MMP24	1.21	-1.21	-0.50
NLN	1.21	-1.21	-0.37
ACAT1	1.20	-1.20	-0.51
ADK	1.19	-1.19	-0.57
TMEM164	1.18	-1.18	-0.81
CYB561D2	1.17	-1.17	-0.58
DTD2	1.17	-1.17	-0.45
FH	1.17	-1.17	-0.45
GSTZ1	1.14	-1.14	-0.39
NIPSNAP1	1.14	-1.14	-0.50
ORMDL2	1.14	-1.14	-0.40
OCIAD2	1.13	-1.13	-0.37
CLN3	1.12	-1.12	-0.43
MRPS16	1.12	-1.12	-0.43
GGT1	1.11	-1.11	-0.51
PAICS	1.11	-1.11	-0.45
SLC27A4	1.10	-1.10	-0.44
OSGIN1	1.09	-1.09	-0.42
SDSL	1.09	-1.09	-0.47
MYO5C	1.08	-1.08	-0.48
MMAB	1.07	-1.07	-0.41
NLRP2	1.05	-1.05	-0.63
PTPN13	1.05	-1.05	-0.58
CDH1	1.02	-1.02	-1.01
CLN6	1.02	-1.02	-0.62
HMBS	1.02	-1.02	-0.45
SLC31A1	1.02	-1.02	-0.43
PPA2	1.01	-1.01	-0.40
THNSL1	1.01	-1.01	-0.37
FGFR3	1.00	-1.00	-0.50
HSD17B8	0.99	-0.99	-0.40
PXMP4	0.99	-0.99	-1.08
TCTA	0.99	-0.99	-0.36
EPS8L2	0.98	-0.98	-0.44
MRPS33	0.98	-0.98	-0.45

Supplementary Fig. 11: Terminal node selection for prize-collecting Steiner forest analysis. Terminal genes include 162 genes present in the IREF interactome that are either upregulated in both SARS-CoV-2 infection and aging or downregulated in both SARS-CoV-2 infection and aging. Each terminal gene is prized with its absolute $\log_2$-fold change between SARS-CoV-2 infected A549-ACE2 cells and normal A549-ACE2 cells. (a) Histogram of prizes for the 162 terminal genes along with descriptive statistics. (b) Table of 75 terminal genes upregulated in both SARS-CoV-2 infection and aging (left) and table of 87 terminal genes downregulated in both SARS-CoV-2 infection and aging, along with prize and $\log_2$ fold change information (also indicated by color).

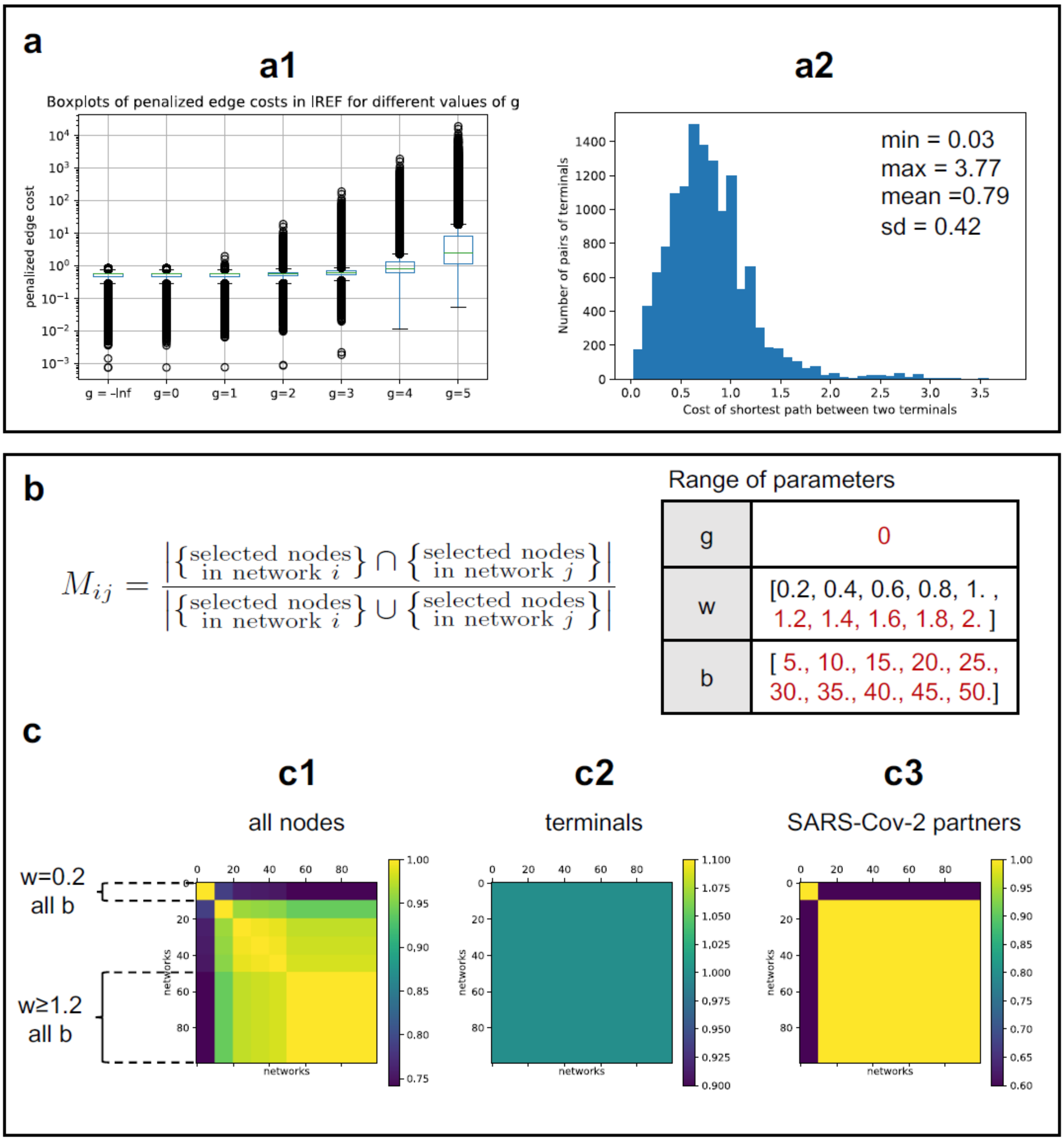

Supplementary Fig. 12: Parameter selection via sensitivity analysis for prize-collecting Steiner forest analysis. (a1) Boxplot of penalized edge costs (log-scale) in the IREF interactome for different values of g. Each boxplot displays the distribution of penalized edge costs for all 182,002 edges of the IREF interactome. The median corresponds to the green middle line, with a box indicating the first (Q1) and third (Q3) quartiles. Whiskers describe the range of the data but do not extend to more than $1.5 \times$ IQR (where IQR=Q3-Q1 denotes the interquartile range). Outliers are plotted as separate dots. The distribution of penalized edge costs is very similar for $g = -\infty$ and $g = 0$. For these values of g, the maximum penalized edge cost is upper bounded by 1. (a2) Histogram of shortest path cost between any two terminals in the IREF interactome for $g = 0$, along with descriptive statistics. The data used in this visualization corresponds to the shortest path cost (computed using Dijkstra's algorithm on the IREF interactome) between all 13,041 unique terminal pairs. (b) Range of parameters g, w and b used in sensitivity analysis. Red values indicate a stable range for the interactome obtained with the prize-collecting Steiner forest algorithm. We retain $g = 0$, $w = 1.4$ and $b = 40$ for our subsequent analysis. (c1-3) Heatmaps of the matrix M indexed for different types of selected nodes: all nodes (c1), terminal nodes (c2) and SARS-CoV-2 interaction partners (c3). Each row/column corresponds to a prize-collecting Steiner forest obtained from a given set of parameters $(g = 0, w, b)$. A stability region for the prize-collection Steiner forest solution appears for $g = 0$, $w \geq 1.2$ and $b \in [5, 50]$.

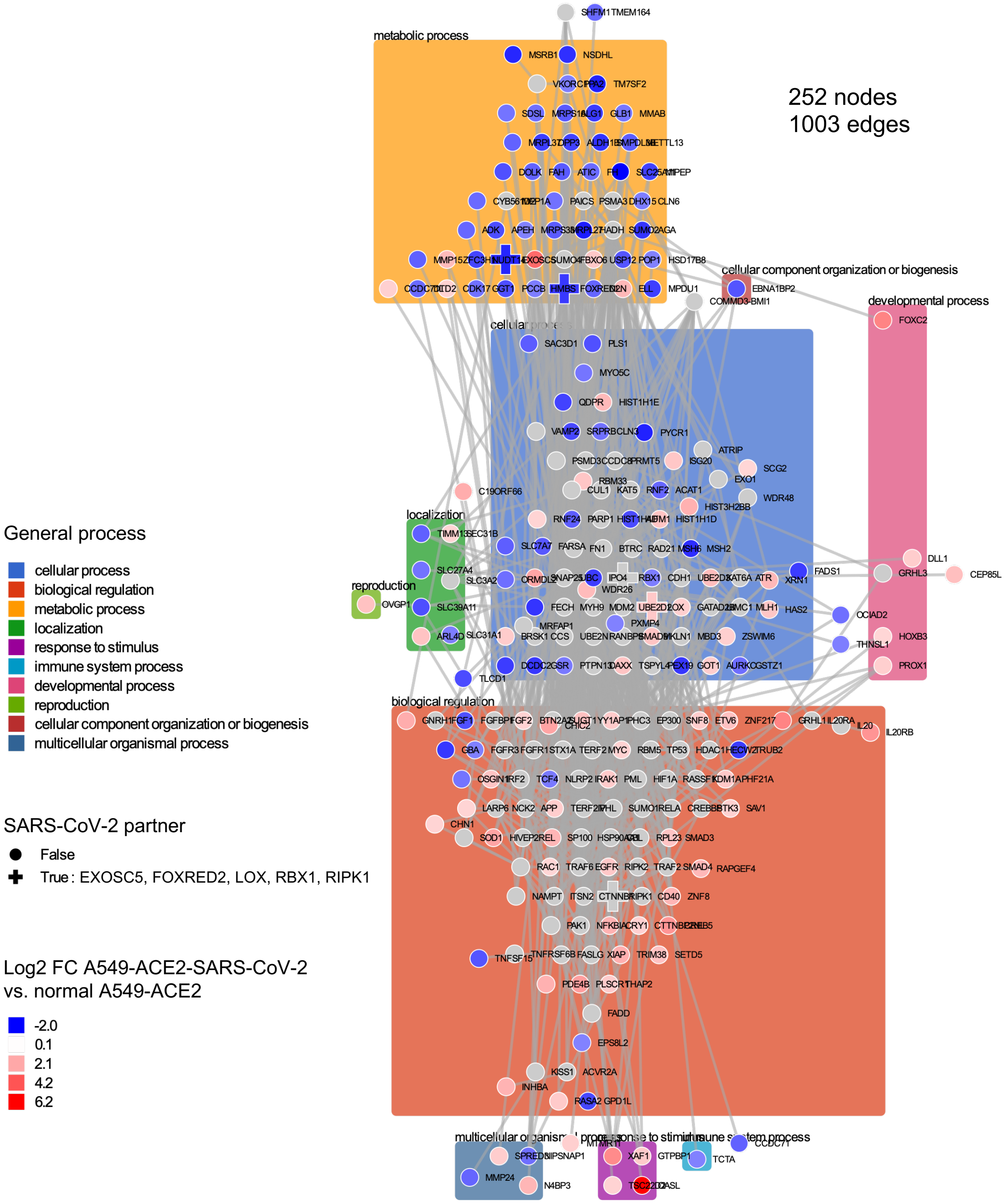

Supplementary Fig. 13: Interactome obtained from the prize-collecting Steiner forest algorithm (with parameters $g = 0$, $w = 1.4$, $b = 40$) using the terminal gene list from Supplementary Fig. 11. The interactome contains 1,003 edges between 252 genes, five of which are known SARS-CoV-2 interaction partners (EXOSC5, FOXRED2, LOX, RBX1, RIPK1, indicated by crosses. Genes in the interactome are grouped by general process (colored boxes in background). Terminal genes are colored by $\log_2$-fold change between SARS-CoV-2-infected and normal A549-ACE2 cells, while Steiner nodes appear in grey.

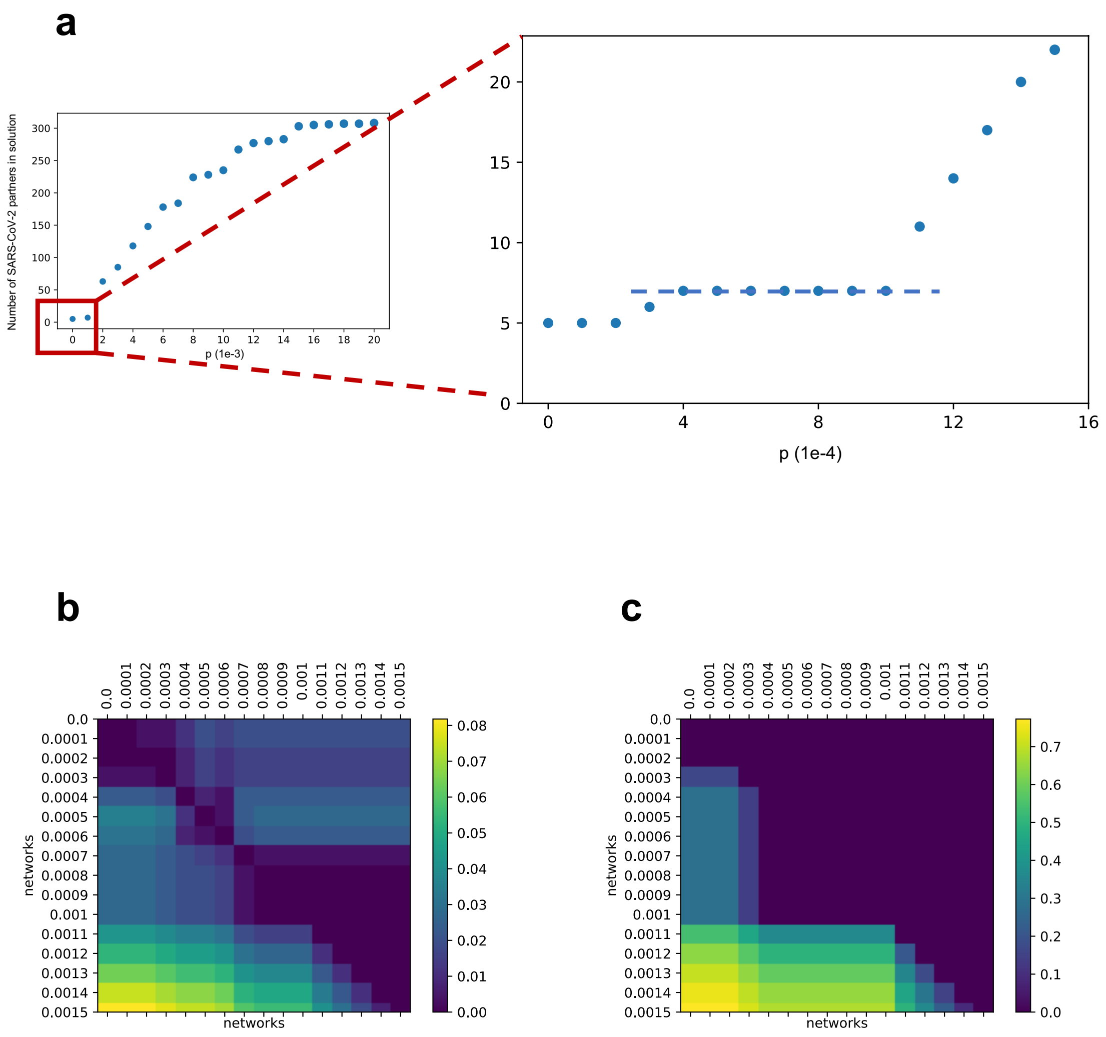

$$M_{ij} = \frac{|\{\text{all nodes in network } i\} \setminus \{\text{all nodes in network } j\}|}{|\{\text{all nodes in network } i\}|}$$

$$M_{ij} = \frac{\left|\left\{\substack{\text{SARS-Cov-2 partners} \\ \text{in network } i}\right\} \setminus \left\{\substack{\text{SARS-Cov-2 partners} \\ \text{in network } j}\right\}\right|}{\left|\left\{\substack{\text{SARS-Cov-2 partners} \\ \text{in network } i}\right\}\right|}$$

Supplementary Fig. 14: Selection of the prize p for non-terminal SARS-CoV-2 interaction partners (all but EXOSC5, FOXRED2 and LOX) via sensitivity analysis. (a) Number of SARS-CoV-2 interaction partners collected in the interactome obtained from the prize-collecting Steiner forest algorithm for different values of p ranging from 0 to 0.02. For $p > 0.02$, all known SARS-CoV-2 interaction partners present in the IREF network are collected in the final interactome. A stability region appears for $p \in [4 \cdot 10^{-4}, 10^{-3}]$ with 7 SARS-CoV-2 interaction partners collected. (b-c) Heatmaps of the matrix M indexed for different types of selected nodes: all nodes (b), and SARS-CoV-2 interaction partners (c). Each row/column corresponds to a prize-collecting Steiner forest obtained from a given set of parameters $(g = 0, w = 1.4, b = 40, p)$. A stability region for the prize-collection Steiner forest solution appears for $g = 0$, $w = 1.4$ and $b = 40$ and $p \in [7 \cdot 10^{-4}, 10^{-3}]$. We retain $g = 0$, $w = 1.4$, $b = 40$ and $p = 8 \cdot 10^{-4}$ for our subsequent analysis.

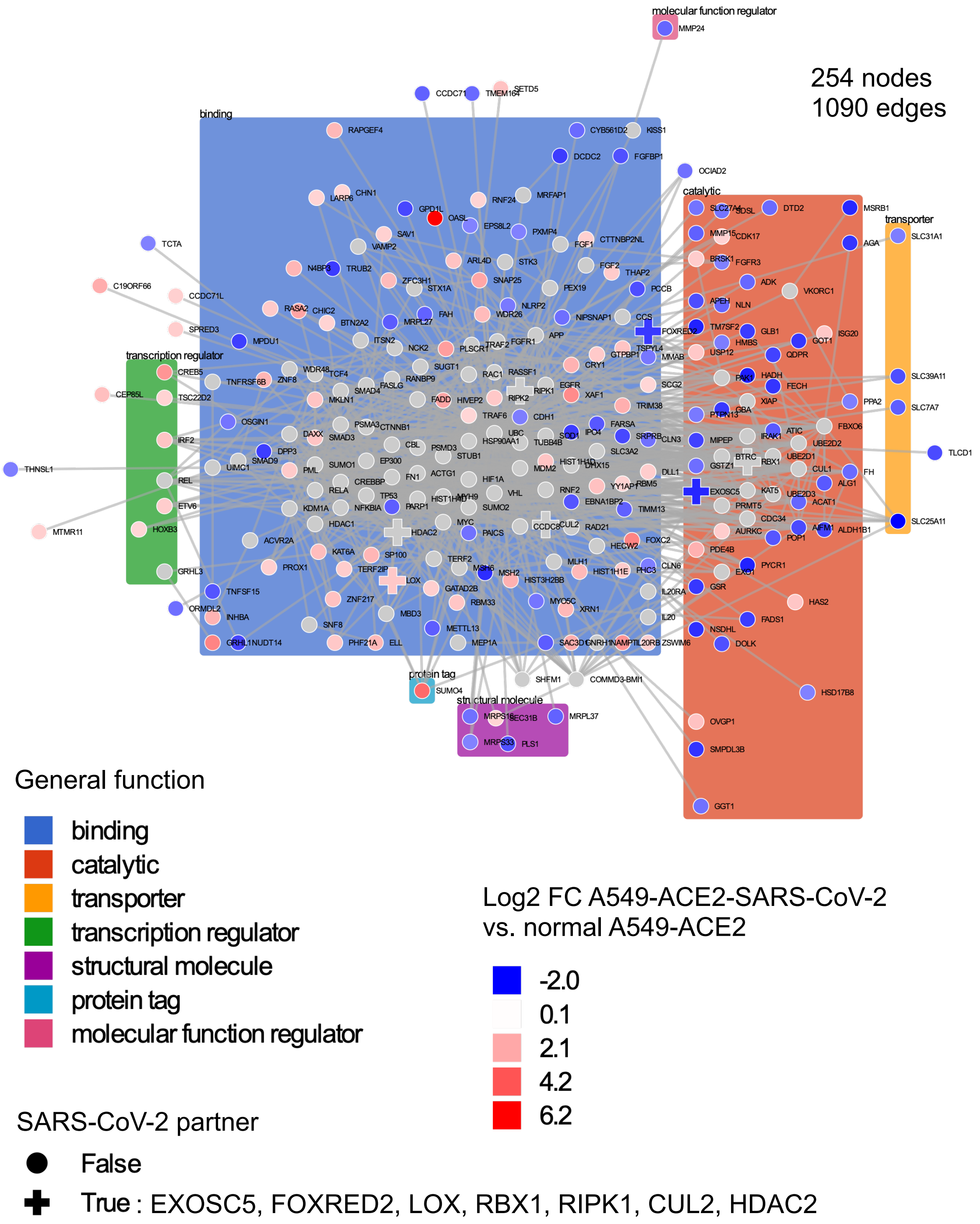

Supplementary Fig. 15: Interactome obtained from the prize-collecting Steiner forest algorithm (with parameters $g = 0$, $w = 1.4$, $b = 40$) using the terminal gene list from Supplementary Fig. 11 augmented with all other SARS-CoV-2 interaction partners prized with $p = 8 \cdot 10^{-4}$. The interactome contains 1,090 edges between 254 genes, seven of which being known SARS-CoV-2 interaction partners (EXOSC5, FOXRED2, LOX, RBX1, RIPK1, CUL2, HDAC2, indicated by crosses). Genes in the interactome are grouped by general function (colored boxes in background). Terminal genes are colored by $\log_2$-fold change between SARS-CoV-2-infected and normal A549-ACE2 cells, while Steiner nodes appear in grey.

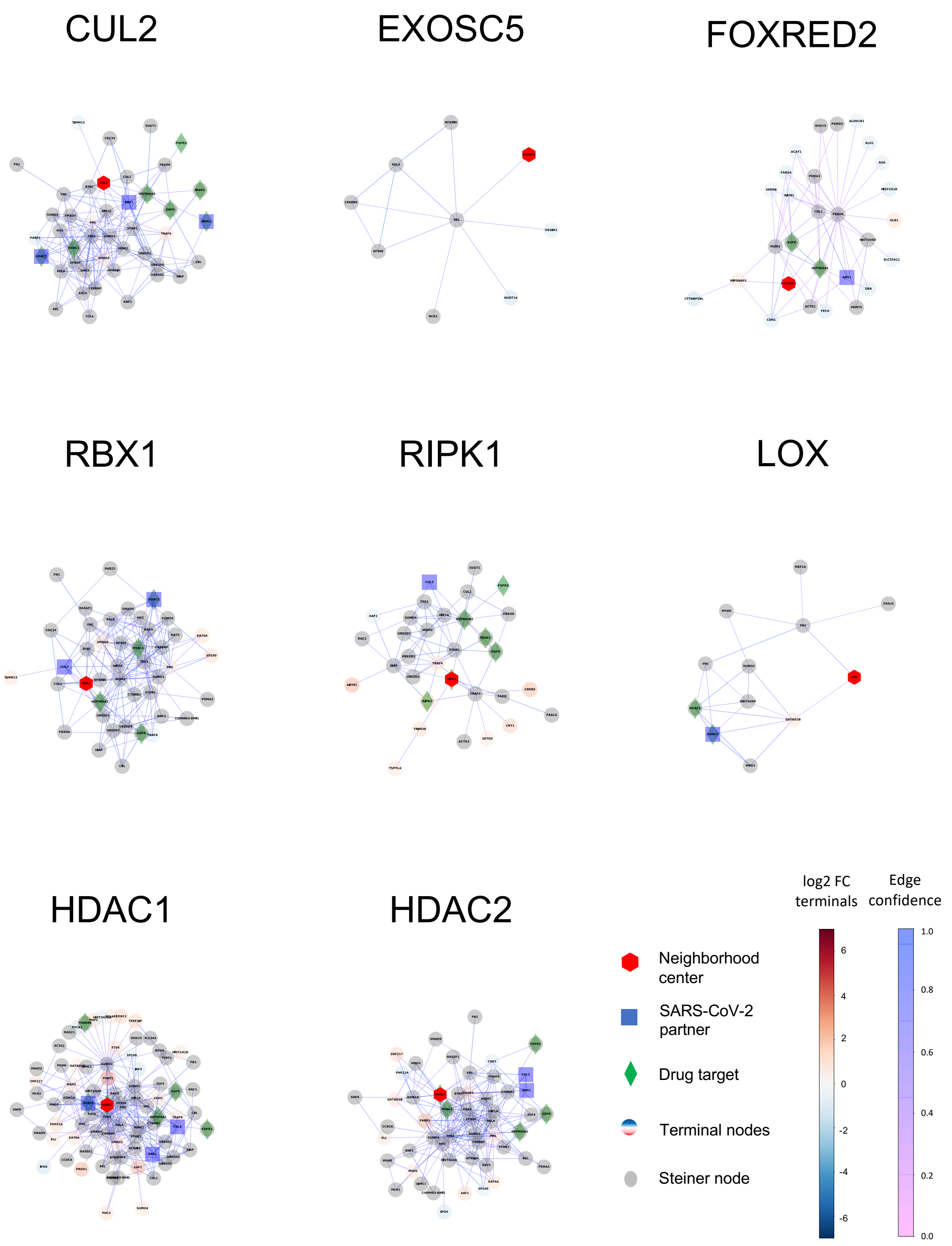

Supplementary Fig. 16: 2-Nearest-Neighborhoods of nodes of interest (denoted by a red hexagon) in the interactome of Supplementary Fig. 15 (parameters $g = 0$, $w = 1.4$, $b = 40$, $p = 8 \cdot 10^{-4}$). Proteins known to interact with SARS-CoV-2 are denoted as blue squares, drug targets are denoted as green diamonds, terminal nodes are colored according to $\log_2$-fold change in SARS-CoV-2-infected A549-ACE2 cells versus normal A549-ACE2 cells, Steiner nodes appear in grey. Edges are colored according to edge confidence, which is thresholded to improve readability (see Methods).

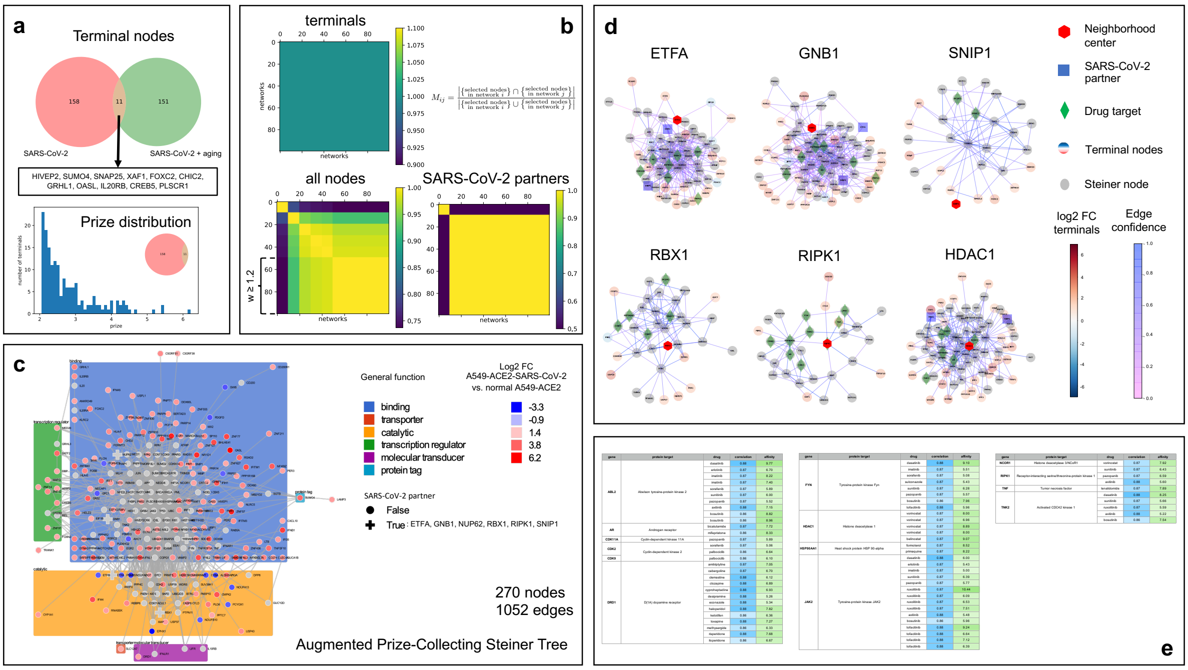

Supplementary Fig. 17: Drug target discovery via prize-collecting Steiner forest analysis to identify putative molecular pathways linking differentially expressed genes in SARS-CoV-2 infection without taking into account age-related differential expression. (a) The general procedure to obtain the interactome is identical to the one described in Fig. 4a, with a different terminal gene list. A histogram of the prize distribution is provided for 169 terminal nodes corresponding to genes differentially expressed in SARS-CoV-2 infection after removing the effect of the ACE2 receptor (red circle and brown intersection). Only 11 of these 169 genes (brown intersection) belong to the terminal nodes list used in Fig. 4 (green circle and brown intersection). The prize of a terminal node equals the absolute value of its $\log_2$-fold change in SARS-CoV-2-infected A549-ACE2 cells versus normal A549-ACE2 cells based on data from [1]. (b) Sensitivity analysis to choose the parameters w and b for the prize-collecting Steiner forest algorithm, including heatmaps of the matrix M indexed for different types of selected nodes: terminal nodes (top), all nodes (bottom left) and SARS-CoV-2 interaction partners (bottom right). Each row/column corresponds to a prize-collecting Steiner forest obtained from a given set of parameters $(g = 0, w, b)$. A stability region for the prize-collection Steiner forest solution appears for $g = 0$, $w \geq 1.2$ and $b \in [5, 50]$. We select $g = 0$, $w = 1.4$ and $b = 40$ corresponding to a robust solution for moderate changes in the parameters. (c) Interactome obtained using the prize-collecting Steiner forest algorithm on the IREF interactome using the terminal genes of Supplementary Fig. 17(a). Proteins are grouped by general function (colored boxes in the background) and marked with a cross if known to interact with SARS-CoV-2 proteins based on data from [2]. (d) 2-Nearest-Neighborhoods of nodes of interest (denoted by a red hexagon) in the interactome. Proteins known to interact with SARS-CoV-2 are denoted as blue squares, drug targets are denoted as green diamonds, terminal nodes are colored according to $\log_2$-fold change in SARS-CoV-2-infected A549-ACE2 cells versus normal A549-ACE2 cells, Steiner nodes appear in grey. Edges are colored according to edge confidence, which is thresholded to improve readability (see Methods). (e) Table of drug targets and corresponding drugs in the interactome. Selected drugs are FDA-approved, high affinity (at least one of the activity constants K_i, K_d, IC50 or EC50 is below $10\mu M$), and match the SARS-CoV-2 signature well (correlation > 0.86). The affinity column displays (and is colored by) $-\log_{10}(\text{activity})$. The correlation column displays (and is colored by) correlations between drug signatures and the reverse signature of SARS-CoV-2 infection based on the overparameterized autoencoder embedding. The protein name corresponding to each gene is included.

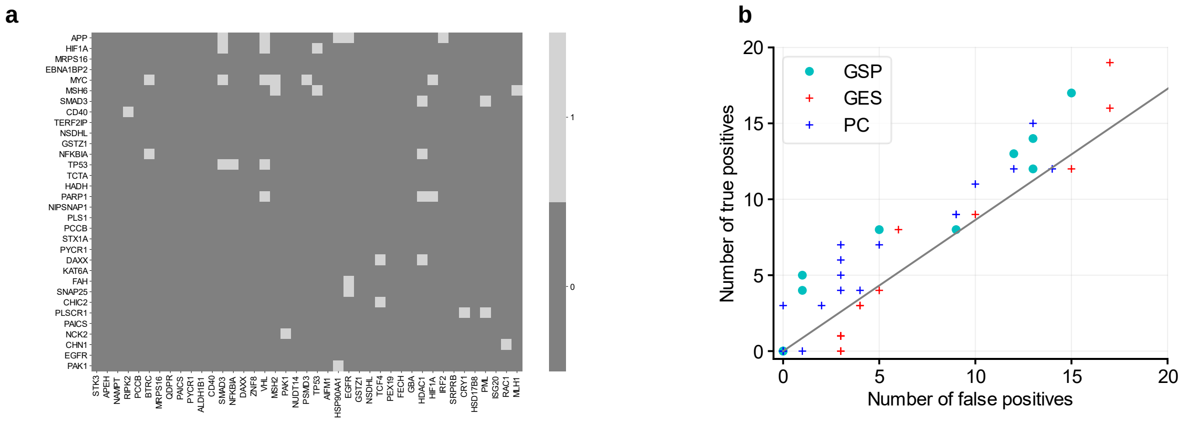

Supplementary Fig. 18: (a) Matrix Q of estimated effects of interventions (columns) on measured genes (rows) in A549 cells from CMap gene knockout and overexpression data with $Q_{ij} = 1$ representing that perturbing gene j affects gene i and hence that gene i is downstream of gene j. (b) ROC curve evaluating causal structure discovery methods GSP (turquoise), PC (blue) and GES (red) for predicting the effects of interventions in A549 cells. The performance of each algorithm is measured by sampling random causal graphs and measuring number of true positives and false positives (see Methods). GSP performs significantly above random guessing with p-value of 0.0177, while PC achieves p-value of 0.0694 and GES a p-value of 0.5867. The grey line represents a random guessing baseline (not used for computation of p-value) based on the number of ground truth positives and negatives, calculated from Q and scaled to extend from $(0, 0)$ to span the entirety of the plot.

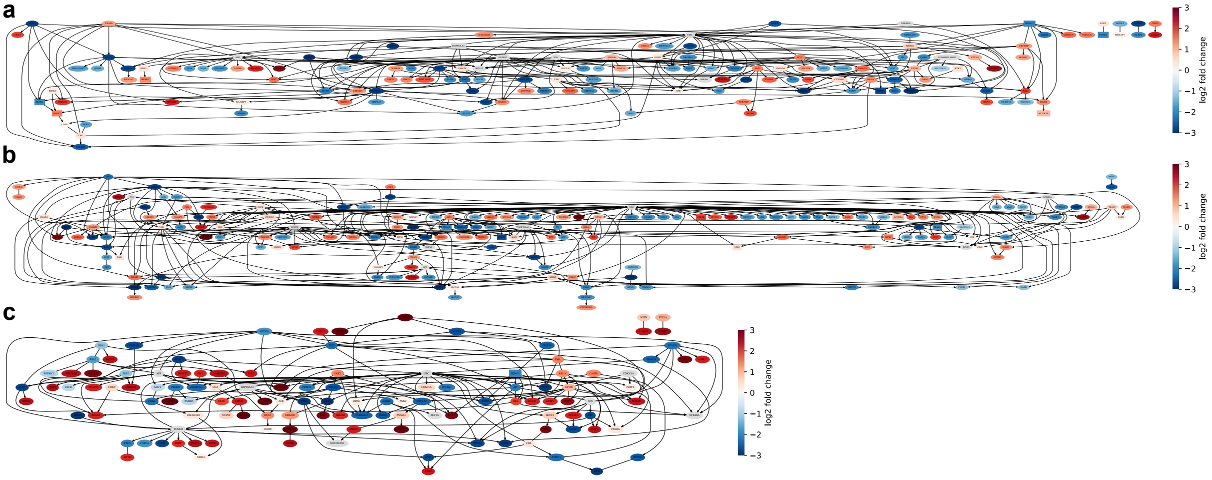

Supplementary Fig. 19: (a) Causal network corresponding to A549 cells. (b) Causal network corresponding to AT2 cells. (c) Causal network corresponding to A549 cells learned using PPI interactome obtained without considering age-associated genes as a prior. All non-singleton nodes are shown, gene targets of drugs selected via our computational drug repurposing pipeline are in boxes and the node color corresponds to the $\log_2$-fold change of expression between A549-ACE2 cells with SARS-CoV-2 infection versus without SARS-CoV-2 infection. Grey nodes represent Steiner nodes.

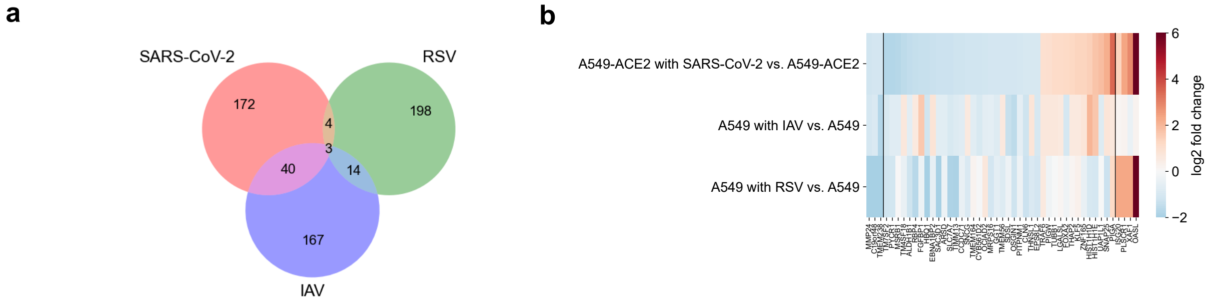

Supplementary Fig. 20: (a) Venn diagram of overlap between differentially expressed genes in SARS-CoV-2 (pink circle), RSV (green circle) and IAV (purple circle) infections. (b) Heatmap of $\log_2$ fold change of differentially expressed genes shared by SARS-CoV-2, IAV and RSV (first 3 genes), SARS-CoV-2 and IAV (40 genes), and SARS-CoV-2 and RSV (last 4 genes).

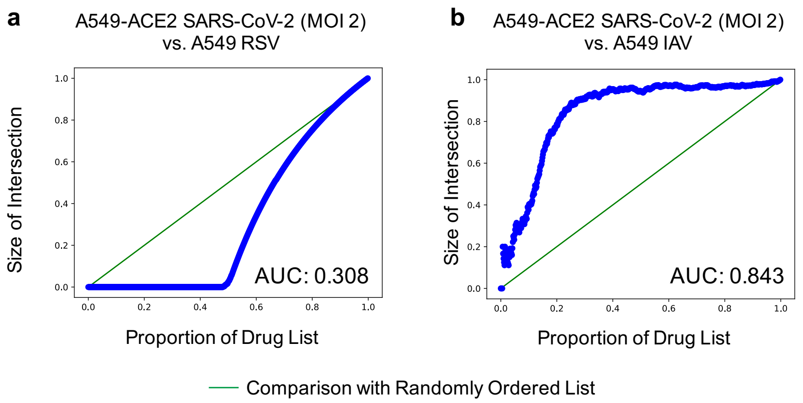

Supplementary Fig. 21: Quantitative analysis of similarity between drug lists obtained using the overparameterized autoencoder on gene expression data from different virus infections. Comparison of drug lists from SARS-CoV-2 infected A549-ACE2 cells versus (a) RSV-infected A549 cells, and (b) IAV-infected A549 cells.

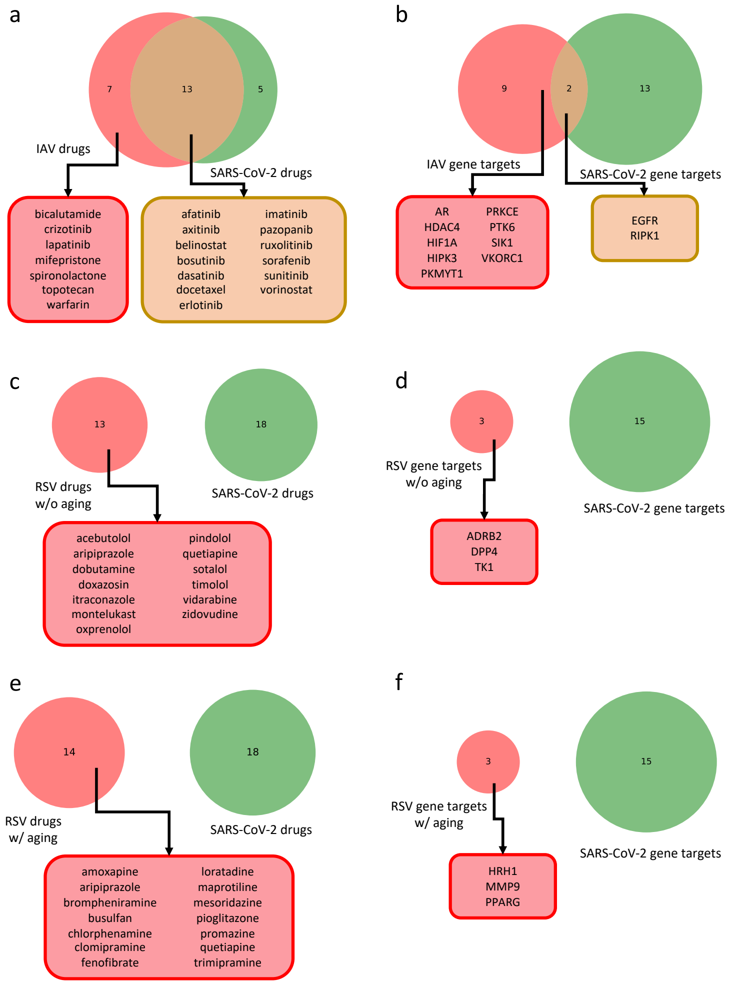

Supplementary Fig. 22: Drugs and their gene targets obtained from the prize-collecting Steiner tree analysis for IAV and RSV in comparison to our findings for SARS-CoV-2. (a) Venn diagram between selected drugs for IAV (red circle) and SARS-CoV-2 (green circle) using aging as a filter in the differential gene expression analysis for both viruses, and (b) Venn diagram for the respective gene targets. (c) Venn diagram between selected drugs for RSV (red circle) and SARS-CoV-2 (green circle) without taking aging into account for the differential expression analysis of RSV, and (d) Venn diagram for the respective gene targets. (e) Venn diagram between selected drugs for RSV (red circle) and SARS-CoV-2 (green circle) using aging as a filter in the differential gene expression analysis for both viruses, and (f) Venn diagram for the respective gene targets.

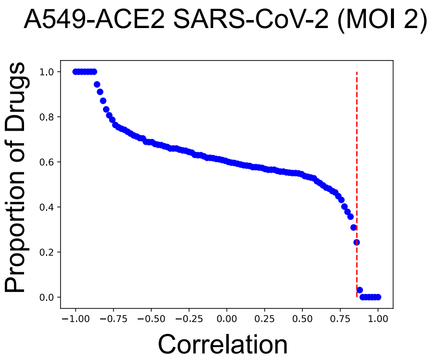

Supplementary Fig. 23: Selection of correlation threshold for identifying candidate drugs. Plot showing the percentage of drugs (y-axis) with correlation higher than a given threshold (x-axis). The vertical red line indicates the x-value (0.86) for which the y-value shows the largest jump and corresponds to the threshold used for the selection of drug candidates.

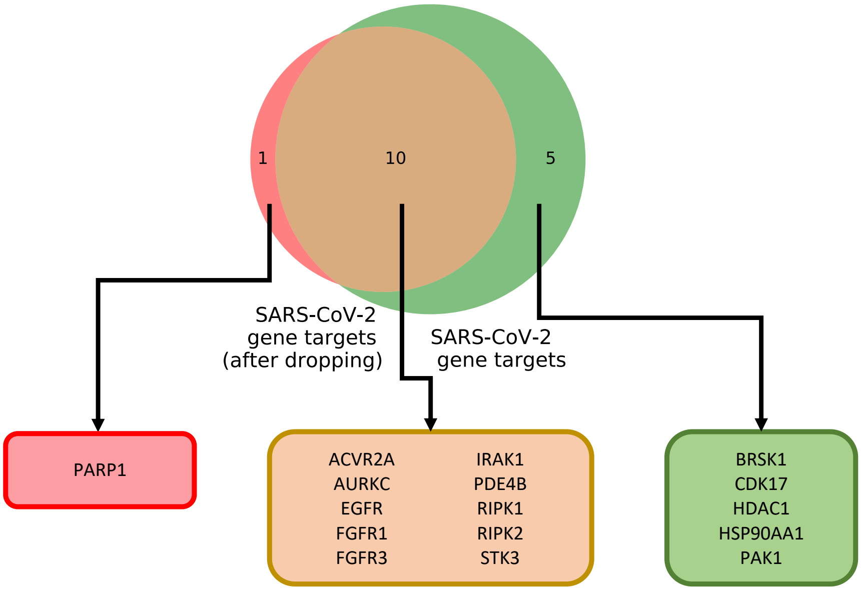

Supplementary Fig. 24: Comparison of drug targets resulting from analyzing the CMap dataset with (red circle) and without removing confounding 1's (green circle).

Supplementary Tables

Drug name	% differentially expressed nodes downstream (A549)	% nodes downstream (A549 no age)	% nodes downstream (AT2)
afatinib	98.51	0.00	83.93
axitinib	98.51	0.85	83.93
bosutinib	98.51	0.00	83.93
dasatinib	98.51	0.00	83.33
erlotinib	98.51	0.00	83.33
imatinib	98.51	0.00	83.93
pazopanib	98.51	0.85	83.93
ruxolitinib	98.51	0.00	83.33
sorafenib	97.01	0.00	0.60
sunitinib	98.51	0.85	83.93
tofacitinib	1.49	0.00	0.00
belinostat	98.51	94.92	83.33
vorinostat	98.51	94.92	83.33
formoterol	98.51	94.92	83.33
primaquine	98.51	94.92	83.33
vardenafil	0.00	0.00	0.00
milrinone	0.00	0.00	0.00
docetaxel	98.51	0.00	83.33

Supplementary Table 1: Percentage of nodes in the largest connected component of the corresponding causal graph that are targeted by each drug. For A549 cells, only genes that are associated with SARS-CoV-2 and aging are considered.

			Frequency of appearance in randomizations			
**drug**	**Selected**	**# targets in PPI**	**Gene labels**	**CMAP signatures**	**Terminal genes**	**PPI network**
sunitinib	1	260	1.0	0.25	0.997	1.0
bosutinib	1	203	0.998	0.24	0.993	1.0
axitinib	1	99	0.997	0.25	0.98	1.0
dasatinib	1	128	0.98	0.246	0.98	1.0
sorafenib	1	116	0.998	0.266	0.975	1.0
pazopanib	1	103	0.991	0.235	0.965	1.0
ruxolitinib	1	132	0.988	0.243	0.94	1.0
erlotinib	1	96	0.967	0.234	0.933	1.0
afatinib	1	38	0.94	0.226	0.863	1.0
vardenafil	1	13	0.348	0.247	0.071	1.0
milrinone	1	9	0.178	0.253	0.034	1.0
imatinib	1	69	0.947	0.238	0.921	0.971
vorinostat	1	32	0.79	0.261	0.8	0.898
belinostat	1	11	0.743	0.225	0.755	0.867
docetaxel	1	13	0.422	0.251	0.576	0.796
tofacitinib	1	43	0.481	0.243	0.58	0.709
formoterol	1	5	0.326	0.253	0.499	0.59
primaquine	1	5	0.344	0.24	0.463	0.555
palbociclib	0	13	0.924		0.741	0.863
mifepristone	0	10	0.634		0.544	0.747
vemurafenib	0	4	0.246		0.393	
danazol	0	16	0.501		0.377	
tacrolimus	0	13	0.418		0.29	
haloperidol	0	42			0.286	
bicalutamide	0	2	0.278		0.277	
clozapine	0	39			0.195	
risperidone	0	36			0.188	
sulconazole	0	25			0.186	
econazole	0	41	0.439		0.164	
amitriptyline	0	33			0.138	
clemastine	0	25			0.103	
dipyridamole	0	19	0.353		0.103	
phentolamine	0	17			0.095	
iloperidone	0	24			0.092	
methysergide	0	22			0.092	
cyproheptadine	0	29			0.09	
carteolol	0	2			0.083	
lenalidomide	0	2			0.083	
cabergoline	0	17			0.079	
loxapine	0	29			0.079	
digitoxin	0	9			0.076	
terconazole	0	17	0.198		0.069	
ketotifen	0	17			0.065	
desipramine	0	22			0.054	
rosuvastatin	0	2			0.054	
perphenazine	0	16			0.053	
naftifine	0	2			0.05	
desoximetasone	0	1			0.048	
flunisolide	0	1			0.048	
halcinonide	0	1			0.048	
irinotecan	0	7			0.048	
phenelzine	0	10			0.048	

prednisone	0	2			0.048	
buspirone	0	13			0.046	
guanfacine	0	8			0.043	
terazosin	0	7			0.039	
sertraline	0	19			0.038	
flumazenil	0	36			0.037	
daunorubicin	0	1			0.036	
bortezomib	0	15		0.241		
caffeine	0	3	0.324			
cisplatin	0	10		0.234		
clofarabine	0	2	0.216			
dobutamine	0	23		0.226		
famotidine	0	3		0.24		
gefitinib	0	72		0.232		
glimepiride	0	4	0.18			
iloprost	0	8	0.206			
lapatinib	0	13		0.256		
midodrine	0	1		0.254		
mitoxantrone	0	18		0.23		
montelukast	0	21		0.251		
nilotinib	0	70		0.292		
olaparib	0	4		0.261		
panobinostat	0	11		0.247		
sildenafil	0	20		0.233		
sitagliptin	0	2	0.183			
tamoxifen	0	51		0.278		
tolbutamide	0	2	0.18			
topotecan	0	5		0.277		
treprostinil	0	6	0.206			
warfarin	0	1		0.254		
zafirlukast	0	13		0.238		

Supplementary Table 2: Frequency of a drug's presence in the list of final drugs after performing Steiner tree analysis with randomization of gene labels, CMap signatures, terminal genes, and the PPI network (1000 randomization runs). Only FDA-approved drugs with high affinity (at least one of the activity constants K_i, K_d, IC50 or EC50 is below $10\mu M$, the drug is dropped if no activity constant is available) and high correlation (> 0.86) with the reverse SARS-CoV-2 signature are considered, amounting to 104 drugs. Green rows correspond to final drugs selected in the non-randomized analysis, while red rows correspond to final drugs selected in the randomized analysis that had not been selected in the non-randomized analysis.

Supplementary References

1. Blanco-Melo, D. *et al.* Imbalanced host response to SARS-CoV-2 drives development of COVID-19. *Cell* (2020).
2. Gordon, D. E. *et al.* A SARS-CoV-2 protein interaction map reveals targets for drug repurposing. *Nature* (2020).
