# Supplementary material for: Causal network models of SARS-CoV-2 expression and aging to identify candidates for drug repurposing
Source: Nat Commun. 2021 Feb 15;12:1024. doi: 10.1038/s41467-021-21056-z (PMC7884845; doi:10.1038/s41467-021-21056-z)
Supplement: Supplementary file 3 — Reporting Summary [file 41467_2021_21056_MOESM3_ESM.pdf]

nature research

Corresponding author(s): Caroline Uhler

Last updated by author(s): Dec 17, 2020

# Reporting Summary

Nature Research wishes to improve the reproducibility of the work that we publish. This form provides structure for consistency and transparency in reporting. For further information on Nature Research policies, see our Editorial Policies and the Editorial Policy Checklist.

## Statistics

For all statistical analyses, confirm that the following items are present in the figure legend, table legend, main text, or Methods section.

| n/a | Confirmed | |
|---|---|---|
| x | | The exact sample size (*n*) for each experimental group/condition, given as a discrete number and unit of measurement |
| x | | A statement on whether measurements were taken from distinct samples or whether the same sample was measured repeatedly |
| | x | The statistical test(s) used AND whether they are one- or two-sided<br>*Only common tests should be described solely by name; describe more complex techniques in the Methods section.* |
| x | | A description of all covariates tested |
| | x | A description of any assumptions or corrections, such as tests of normality and adjustment for multiple comparisons |
| x | | A full description of the statistical parameters including central tendency (e.g. means) or other basic estimates (e.g. regression coefficient) AND variation (e.g. standard deviation) or associated estimates of uncertainty (e.g. confidence intervals) |
| | x | For null hypothesis testing, the test statistic (e.g. $F$, $t$, $r$) with confidence intervals, effect sizes, degrees of freedom and $P$ value noted<br>*Give P values as exact values whenever suitable.* |
| x | | For Bayesian analysis, information on the choice of priors and Markov chain Monte Carlo settings |
| x | | For hierarchical and complex designs, identification of the appropriate level for tests and full reporting of outcomes |
| | x | Estimates of effect sizes (e.g. Cohen's $d$, Pearson's $r$), indicating how they were calculated |

*Our web collection on statistics for biologists contains articles on many of the points above.*

## Software and code

Policy information about availability of computer code

| | |
|---|---|
| Data collection | No software was used for data collection. |
| Data analysis | R (v3.6) package pcalg (v2.6), python (v3.7) packages OmicsIntegrator2 (v2) causaldag (v0.1a133), GSEApy (v0.9.18), networkx (v2.4), numpy (v1.17.3), pandas (v0.25.3), PyTorch (v1.6), scikit-learn (v0.22.2), scipy (v1.4.1), cmapPy (v4.0.1), graphviz (v2.40.1), https://github.com/uhlerlab/covid19_repurposing (v1.0) |

For manuscripts utilizing custom algorithms or software that are central to the research but not yet described in published literature, software must be made available to editors and reviewers. We strongly encourage code deposition in a community repository (e.g. GitHub). See the Nature Research guidelines for submitting code & software for further information.

## Data

Policy information about availability of data

All manuscripts must include a data availability statement. This statement should provide the following information, where applicable:

- Accession codes, unique identifiers, or web links for publicly available datasets
- A list of figures that have associated raw data
- A description of any restrictions on data availability

All datasets used in this work are publicly available from the following sources: The gene expression data for SARS-CoV-2 was obtained from GSE147507 (https://www.ncbi.nlm.nih.gov/geo/query/acc.cgi?acc=GSE147507) [23] and the gene expression data for the aging analysis was obtained from https://gtexportal.org/home/index.html [24]. The CMap data was downloaded using accession code GSE92742 (https://www.ncbi.nlm.nih.gov/geo/query/acc.cgi?acc=GSE92742) [2]. We used the PPI network from http://github.com/fraenkel-lab/OmicsIntegrator2 (IRefIndex Version 14) [42] and drug target data from DrugCentral (http://drugcentral.org/download) [44, 45]. The single-cell RNA-seq data for the causal analysis was obtained from GSE81861 (https://www.ncbi.nlm.nih.gov/geo/query/acc.cgi?acc=GSE81861) [46] for A549 cells and http://www.nupulmonary.org/resources for AT2 cells associated with [49]. The host-pathogen interactions of SARS-

CoV-2 proteins were obtained from http://www.ndexbio.org/#/network/5d97a04a-6fab-11ea-bfdc-0ac135e8bacf [6].

# Field-specific reporting

Please select the one below that is the best fit for your research. If you are not sure, read the appropriate sections before making your selection.

- [x] Life sciences
- [ ] Behavioural & social sciences
- [ ] Ecological, evolutionary & environmental sciences

For a reference copy of the document with all sections, see nature.com/documents/nr-reporting-summary-flat.pdf

# Life sciences study design

All studies must disclose on these points even when the disclosure is negative.

| | |
|---|---|
| Sample size | Since all datasets used in this work were from previously published sources, the sample sizes were also obtained from these sources (listed in data availability statement). |
| Data exclusions | Relevant subsets of data were used for each type of analysis (detailed in Methods and main text). We excluded genes with NA or inf values from analysis. A cluster of points in the CMap dataset corresponding to a batch effect (based on minimum gene expression value) was removed via k-means. For single-cell analysis genes displaying high dropout rate were removed. For single-cell AT2 RNA-seq data only the data from Donor 7 (chosen based on largest number of cells) was analyzed to avoid batch effects. |
| Replication | We compare the findings of our method on two different batches of data, on different cell types (A549 vs. A549-ACE2) and on three different viruses (SARS-CoV-2, IAV, RSV). |
| Randomization | We performed randomization of various inputs (PPI network, gene expression data, CMap signatures, terminal nodes) as described in the Supplementary Note. |
| Blinding | Since effective drugs or drug targets are not currently known against SARS-CoV-2 with high certainty, no blinding was necessary. |

# Reporting for specific materials, systems and methods

We require information from authors about some types of materials, experimental systems and methods used in many studies. Here, indicate whether each material, system or method listed is relevant to your study. If you are not sure if a list item applies to your research, read the appropriate section before selecting a response.

## Materials & experimental systems

| n/a | Involved in the study |
|---|---|
| [x] | [ ] Antibodies |
| [x] | [ ] Eukaryotic cell lines |
| [x] | [ ] Palaeontology and archaeology |
| [x] | [ ] Animals and other organisms |
| [x] | [ ] Human research participants |
| [x] | [ ] Clinical data |
| [x] | [ ] Dual use research of concern |

## Methods

| n/a | Involved in the study |
|---|---|
| [x] | [ ] ChIP-seq |
| [x] | [ ] Flow cytometry |
| [x] | [ ] MRI-based neuroimaging |
