# Supplementary material for: Causal network models of SARS-CoV-2 expression and aging to identify candidates for drug repurposing
Source: Nat Commun. 2021 Feb 15;12:1024. doi: 10.1038/s41467-021-21056-z (PMC7884845; doi:10.1038/s41467-021-21056-z)
Supplement: Supplementary file 5 — Description of Additional Supplementary Files [file 41467_2021_21056_MOESM5_ESM.pdf]

Title: Supplementary Data 1

Description: Correlation of each drug applied to A549, MCF7 and HCC515 cells measured in CMap with the direction from SARS-CoV-2 infection to normal in A549-ACE2 cells, calculated using autoencoder embedding, original space and top 100 principal components
